# Prophylactic Biliary Stenting After Stone Clearance Improves the Safety of Needle‐Knife Fistulotomy: A Propensity Score‐Matched Analysis

**DOI:** 10.1002/jgh3.70244

**Published:** 2025-07-30

**Authors:** Amir Sadeghi, Erfan Arabpour, Mohammad Abdehagh, Mohammad Reza Zali

**Affiliations:** ^1^ Gastroenterology and Liver Diseases Research Center, Research Institute for Gastroenterology and Liver Diseases Shahid Beheshti University of Medical Sciences Tehran Iran

**Keywords:** biliary stent, endoscopic retrograde cholangiopancreatography, needle‐knife fistulotomy, post‐ERCP pancreatitis

## Abstract

**Objectives:**

Needle‐knife fistulotomy is an advanced technique for gaining biliary access in endoscopic retrograde cholangiopancreatography (ERCP). This study assesses the hypothesis of whether biliary stenting after needle‐knife fistulotomy could improve the safety of the ERCP.

**Methods:**

A retrospective review of the medical records of patients who referred for ERCP between 2021–2024 was performed. All patients with naïve papilla and choledocholithiasis who underwent needle‐knife fistulotomy were included in the study. Patients were categorized into the two groups of with and without biliary stent.

**Results:**

Of the 402 included patients, 331 had successful stone extraction, among whom 50 (15.1%) and 281 (84.9%) recieved and not received a biliary stent, respectively. After 1:4 propensity score matching, age, sex, difficult cannulation, and pancreatic duct cannulation were comparable between the groups (*p* > 0.05). Patients with biliary stent had a borderline significant lower rate of post‐ERCP pancreatitis (2.3% vs. 13.1%, *p* = 0.054). No delayed perforation was observed in either groups. There were no significant differences in cholangitis and bleeding between the groups (*p* > 0.05).

**Conclusions:**

This is the first study investigating the impact of prophylactic biliary stenting after needle‐knife fistulotomy, suggesting that prophylactic biliary stenting after needle‐knife fistulotomy and successful stone removal may improve the safety of ERCP by reducing the rate of post‐ERCP pancreatitis. Further large‐scale prospective studies are warranted to validate these findings.

## 1 Introduction

Endoscopic retrograde cholangiopancreatography (ERCP) is the primary tool for managing common bile duct (CBD) stones. After successful biliary cannulation, stones are extracted using a balloon or basket [1]. However, stone extraction is reported to fail in up to 25% of patients [2]. In cases where stone extraction fails, guidelines recommend placing a biliary stent to establish biliary drainage and facilitate easier stone removal in subsequent attempts [3, 4].

Achieving selective biliary cannulation is essential for the extraction of CBD stones. The conventional transpapillary technique, involving the cannulation of the bile duct via the ampulla of Vater, followed by sphincter incision using electrocautery, has been reported to fail [5]. In challenging cannulation cases, when the standard approach fails, the procedure may take longer, and repeated manipulation of the papilla can increase the risk of adverse events [6]. Precut techniques have been utilized as an alternative means of gaining biliary access. One such technique is needle knife fistulotomy (NKF), which can be performed either as a primary access or rescue technique [7, 8]. Due to the reduced thermal injury to the pancreatic orifice and lower rate of inadvertent pancreatic duct (PD) cannulation with this technique, NKF is becoming increasingly popular due to its lower rate of post‐ERCP pancreatitis (PEP). However, given that this technique involves making incisions in the periampullary region, there is a concern regarding early and delayed perforation; therefore, it is recommended that this technique be performed by experienced endoscopists [9].

Some endoscopists believe that inserting a biliary stent after NKF could improve the safety of the procedure, lower the rate of adverse events, and decrease the rate of delayed biliopancreatic perforations. Nonetheless, to date, there is no study that evaluates this hypothesis and investigates the outcomes of biliary stenting after NKF. This study aims to investigate the impact of biliary stenting on the outcomes and adverse events of ERCP after using NKF for biliary access to extract CBD stones.

## 2 Patients and Methods

### 2.1 Study Design

This study is a retrospective analysis of a prospective ERCP registry of consecutive patients referred for ERCP from April 2021 to September 2024 to the ERCP unit of Taleghani Hospital, a tertiary referral center for pancreatobiliary disorders, Tehran, Iran. During this period, all patients were required to undergo ERCP if they had choledocholithiasis on the basis of physical and paraclinical examinations. All patients with naïve major duodenal papilla (MDP) and CBD stone who underwent NKF as the cannulation approach were considered for inclusion in this study. The exclusion criteria were as follows: [1] age under 18 years, [2] pregnancy or breastfeeding, [3] surgically altered upper gastrointestinal anatomy, [4] chronic pancreatitis, [5] suspected sphincter of Oddi dysfunction, [6] periampullary diverticulum, [7] ampullary tumor, [8] planned for PD cannulation, [9] at least two inadvertent PD cannulation/opacification, [10] unsuccessful deep biliary cannulation, and [11] patients with metal biliary stent.

This study was performed in accordance with the Declaration of Helsinki, and the ethics committee of Shahid Beheshti University of Medical Sciences (SBMU) approved the study with the ethical code IR.SBMU.RIGLD.REC.1398.043. None of the authors had access to information that could identify individual participants during or after data collection.

### 2.2 ERCP Procedures

All of the procedures were conducted by three experienced endoscopists who perform more than 500 therapeutic ERCPs annually (with at least 100 NKFs per year) and have > 90% successful selective biliary cannulation via fistulotomy techniques. No trainee was involved in the procedures.

A 100 mg rectal diclofenac or indomethacin was given to all patients immediately before the operation. Prior to each procedure, benzodiazepines and opioid agents were routinely administered as premedications. The dose was modified according to the patient's condition. A side‐view therapeutic duodenoscope (JF‐240 or JF‐260V; Olympus, Tokyo, Japan) was employed for the ERCP procedure. All patients were hydrated with either normal saline or lactated Ringer solutions following the standard protocol (1.5 mL/kg/h during and for 8 h post‐procedure). A 7‐or 10‐french plastic biliary stent with a length of 7 cm (Advanix, Boston Scientific) was inserted by the preference of the endoscopist. No prophylactic or therapeutic PD stent was placed.

### 2.3 Cannulation Techniques and Stone Extraction

Fistulotomy was performed using a needle‐knife (MicroKnife XL, Boston Scientific) with a pure cutting current, adhering to a standardized protocol. The needle was inserted approximately 3–4 mm from the sheath and aimed at the junction of the upper one‐third and lower two‐thirds of the papillary roof. A superficial incision was created with the needle‐knife at the apex of the infundibulum, located at least 5 mm above the ampullary orifice, oriented in the 11–12 o'clock direction and about 2–3 mm beneath the mucosal surface. The incision was progressively extended until bile was detected, maintaining a minimum distance of 5 mm from the papillary orifice. Once bile was visualized, the fistula was carefully probed with a guidewire to achieve successful cannulation of the CBD. In instances where deep cannulation was not accomplished, the endoscopist executed one or two additional minor yet deeper incisions along the anticipated trajectory of the CBD.

After achieving deep biliary cannulation, all patients underwent biliary sphincterotomy before stone clearance. Additionally, endoscopic papillary balloon dilation was performed at the discretion of the endoscopist. Following the cholangiogram confirmation of a CBD stone, the stone was cleared using a balloon extraction technique. Complete stone clearance was confirmed based on an occlusion cholangiogram.

### 2.4 Patients Follow‐Up

Patients were observed for clinical symptoms suggestive of immediate adverse events, including abdominal pain, local tenderness, emphysema, bleeding, respiratory distress, and loss of consciousness. Vital signs and clinical symptoms were monitored in the recovery room for at least 6 h through the reversing stages of anesthesia. All patients were closely checked for hemoglobin, amylase, and lipase levels. In the absence of significant signs and symptoms, the patients were discharged, and they were trained extensively on the alarm signs. In cases of PEP, bleeding, cholangitis, perforation, or any other significant complaint, patients were needed to stay in the hospital for a longer period. A follow‐up telephone call was made to patients 30 days after discharge to inquire about any delayed adverse events.

For patients with therapeutic biliary stents, stent removal was performed in accordance with European Society of Gastrointestinal Endoscopy (ESGE) guideline [10]. In patients with prophylactic biliary stents, the stent was removed via duodenoscopy 3 days after placement.

### 2.5 Definitions and Classifications

The MDP morphology was defined on the basis of Harraldsson classification [11]. Primary NKF was defined as using NKF as the initial cannulation technique prior to any other cannulation technique, while late NKF was defined as applying NKF after failure of standard transpapillary technique. The cannulation approach was deemed successful if the guidewire entered the CBD, while failure was defined as the inability to cannulate CBD using the chosen method within five number of attempts. Difficult biliary cannulation was characterized according to the ESGE statement (5‐5‐2 definition): (I) > 5 min of cannulation attempts, or (II) > 5 contacts with the papilla, or (III) ≥ 2 inadvertent PD cannulation/opacification [12].

PEP was defined as onset of new or exacerbated abdominal pain attributable to acute pancreatitis, followed by an elevated pancreatic enzyme at least three times the upper limit of normal, leading to prolongation of hospitalization after ERCP. Based on Atlanta criteria, PEP severity was stratified into the mild, moderate, and severe groups [13]. Bleeding was defined by the criteria proposed by Cotton et al. [14], where mild bleeding was characterized by clinical signs of hemorrhage, hemoglobin drop < 3 g/dL, and no need to blood transfusion. Cholangitis was defined based on the Tokyo guidelines in addition to no evidence of acute cholangitis in the week preceding the ERCP procedure [15, 16]. Perforation was characterized as the occurrence of retroperitoneal air or contrast leakage into the retroperitoneal space via an unintended pathway formed as a result of a sphincterotomy or fistulotomy.

### 2.6 Statistical Analysis

All of the analyses were performed using the STATA version 17.0 (StataCorp, College Station, Lakeway, TX, USA) and Python 3.12.7 (Python Software Foundation). Continuous variables were compared with the Student's t‐test or Mann–Whitney U‐test, while categorical variables were compared with Pearson's chi‐square or Fisher's exact tests. Adjustment was performed for the baseline variables that were significantly different between the two groups. The relationship between numerical variables was examined using Pearson's correlation coefficient. Binary logistic regression was used to determine univariate and multivariate odds ratios (OR) with a 95% confidence interval (CI). Log binomial regression was used to determine risk ratio (RR) with a 95% CI. A two‐tailed *p*‐value < 0.05 was considered statistically significant.

### 2.7 Propensity Score Matching

To reduce confounding, we employed propensity score matching (PSM) to estimate the effect of biliary stenting on the outcomes of ERCP. The PSM process involved matching patients who underwent biliary stenting with those who did not, based on their propensity scores. To analyze the outcomes of all cases for PSM, we first selected age, sex, CBD diameter, cannulation technique, difficult cannulation, PD cannulation, and papilla morphology. However, for PSM in successful stone extraction cases, we only selected age, sex, CBD diameter, cannulation technique, difficult cannulation, and PD cannulation due to the small number of patients. A logistic regression model was used to estimate propensity scores, and a nearest neighbor matching algorithm (with 1:2 and 1:4 ratios for all cases and successful stone extraction cases, respectively) was applied within a 0.2 standard deviation caliper of propensity scores.

## 3 Results

**FIGURE 1:**
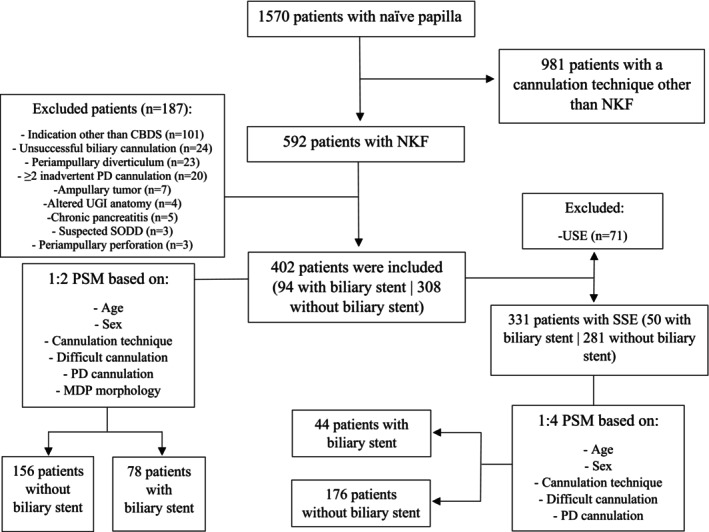
Algorithm presenting an overview of study and matching process. CBDS, common bile duct stone; MDP, major duodenal papilla; NKF, needle‐knife fistulotomy; PD, pancreatic duct; PSM, propensity score matching; SODD, sphincter of oddi dysfunction; SSE, successful stone extraction; UGI, upper gastrointestinal; USE, unsuccessful stone extraction.

Among the 1570 patients with naïve MDP, 592 patients underwent NKF as the cannulation technique; finally, 402 patients met the eligibility criteria and were included in the study. This population was investigated in two cohorts: (I) All of the patients regardless of the stone extraction success (and a subsequent 1:2 PSM), and (II) patients with complete successful stone extraction (and a subsequent 1:4 PSM) (Figure 1).

### 3.1 Cohort I (Patients With CBD Stone Undergoing NKF)

This cohort initially comprised 402 patients (94 with biliary stent, 308 without). Following 1:2 PSM, both groups consisted of 234 patients (78 with biliary stent, 156 without).

Table 1 shows the baseline characteristics, ERCP findings, and the adverse events in this cohort, before and after PSM. Prior to PSM, the stented group was significantly older (59.7 years vs. 55.4 years; *p* = 0.029). This difference was not statistically significant after matching (*p* = 0.556). Sex and body mass index (BMI) were comparable between groups both before and after matching (*p* > 0.05). Papilla morphology also showed no statistically significant differences between groups before or after PSM. Late NKF was more frequent in the stented group before matching (56.4% vs. 36.4%; *p* = 0.001); however, this difference was not observed after matching (53.9% vs. 53.8%; *p* = 1.000). Before matching, the stented group exhibited significantly longer cannulation times (246 s vs. 198 s; *p* < 0.001) and a higher proportion of difficult cannulations (58.5% vs. 40.6%; *p* = 0.002). After matching, these differences were no longer statistically significant for cannulation time (*p* = 0.239) and difficult cannulation (*p* = 0.926). The CBD diameter was larger in the stented group before matching (13.3 vs. 11.3 mm; *p* < 0.001), but this difference was not significant after matching (*p* = 0.906). Balloon dilation was less frequent in the stented group both before (85.1% vs. 95.1%; *p* = 0.001) and after matching (85.9% vs. 94.9%; *p* = 0.018). The presence of CBD sludge and stone number were similar between groups both before and after matching (*p* > 0.05). Before matching, the stented group had larger stones (12.5 mm vs. 8.9 mm; *p* < 0.001); after matching, this difference remained borderline significant (*p* = 0.055). The overall ERCP duration was longer in the stented group before matching, with a higher proportion of procedures exceeding 30 min (43.6% vs. 23.7%; *p* < 0.001). After matching, ERCP durations were similar between groups (*p* = 0.460).

**TABLE 1:** Demographics, ERCP findings, interventions, and adverse events in patients with choledocholithiasis.

Variables	Before PS matching			After PS matching		
	With stent (*n* = 94)	Without stent (*n* = 308)	*p*	With stent (*n* = 78)	Without stent (*n* = 156)	*p*
Age (years)	59.7 ± 15.9	55.4 ± 16.9	0.029	56.8 ± 15.0	58.1 ± 15.4	0.556
Female sex	53 (56.4)	166 (53.9)	0.672	45 (57.7)	86 (55.1)	0.710
BMI (kg/m^2^)	25.3 ± 4.2	25.9 ± 4.9	0.281	25.5 ± 4.4	25.6 ± 5.2	0.954
Papilla morphology						
Regular	23 (35.4)	94 (30.5)	0.072	21 (26.9)	44 (28.2)	0.746
Small	1 (2.3)	3 (1.0)		1 (1.3)	2 (1.3)	
Protruding	13 (11.2)	70 (22.7)		10 (12.8)	30 (19.2)	
Creased	57 (51.1)	141 (45.8)		46 (59.0)	80 (51.3)	
NKF technique						
Primary	41 (43.6)	196 (63.6)	0.001	36 (46.1)	72 (46.2)	1.000
Late	53 (56.4)	112 (36.4)		42 (53.9)	84 (53.8)	
Cannulation time (seconds)	246 ± 110	198 ± 102	< 0.001	245 ± 110	227 ± 107	0.239
Difficult cannulation	55 (58.5)	125 (40.6)	0.002	43 (55.1)	87 (55.8)	0.926
CBD diameter (mm)	13.3 ± 3.9	11.3 ± 3.1	< 0.001	12.3 ± 3.2	12.3 ± 3.1	0.906
Balloon dilation	80 (85.1)	293 (95.1)	0.001	67 (85.9)	148 (94.9)	0.018
CBD sludge	30 (31.9)	93 (30.2)	0.751	28 (35.9)	45 (28.9)	0.272
CBD stone	57 (32.0)	214 (64.7)	< 0.001	56 (47.1)	65 (54.6)	0.243
Stone size (mm)	12.5 ± 7.3	8.9 ± 4.1	< 0.001	11.2 ± 7.5	9.3 ± 4.3	0.055
Stone number	2.5 ± 1.97	2.5 ± 2.1	0.962	2.4 ± 2.0	2.7 ± 2.0	0.440
PD cannulation	28 (29.8)	62 (20.1)	0.049	22 (28.2)	45 (28.9)	0.919
Duration of ERCP						
≤ 15 min	37 (39.4)	182 (59.1)	< 0.001	31 (39.7)	71 (45.5)	0.460
15–30 min	16 (17.0)	53 (17.1)		14 (18.0)	32 (20.5)	
> 30 min	41 (43.6)	73 (23.7)		33 (42.3)	53 (34.0)	
Adverse events						
PEP						
Overall	8 (8.5)	37 (12.0)	0.346	8 (10.3)	21 (13.5)	0.483
Mild	6 (6.4)	27 (8.8)		6 (7.7)	16 (10.3)	
Moderate	2 (2.1)	9 (2.9)		2 (2.6)	5 (3.2)	
Severe	0	1 (0.3)		0	0	
Bleeding	2 (2.1)	1 (0.3)	0.138	2 (2.6)	0	0.110
Cholangitis	2 (2.1)	2 (0.6)	0.234	2 (2.6)	2 (1.3)	0.858
Perforation	0	0	1.000	0	0	1.000
Stent migration	2 (2.1)	0	0.083	2 (2.6)	0	0.110

Abbreviations: BMI, body mass index; CBD, common bile duct; ERCP, endoscopic retrograde cholangiopancreatography; NKF, needle‐knife fistulotomy; PD, pancreatic duct; PEP, post‐ERCP pancreatitis; PS, propensity score.

Adverse event rates did not differ significantly between groups before or after matching. PEP occurred in 8.5% of the stented group versus 12.0% of the non‐stented group before matching (*p* = 0.346) and in 10.3% versus 13.5% after matching (*p* = 0.483). There were no significant differences in bleeding and cholangitis rates between the groups, both before and after PSM (*p* > 0.05). Two stent migrations were observed in the stented group (2.1% before PSM, 2.6% after PSM). No perforations were observed in either group.

**FIGURE 2:**
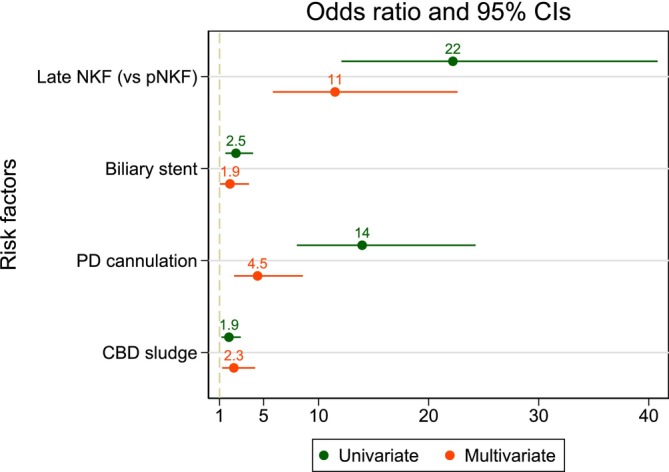
Forest plot of possible risk factors of prolongation of the procedure. CBD, common bile duct; CI, confidence interval; NKF, needle‐knife fistulotomy; PD: pancreatic duct.

Table 2 presents the risk factors of prolongation of procedure based on logistic regression analysis. In univariate analysis, late NKF (OR 22.2, 95% CI 12.08–40.80, *p* < 0.001), CBD sludge (OR 1.85, 95% CI 1.17–2.92, *p* = 0.008), PD cannulation (OR 13.95, 95% CI 8.03–24.26, *p* < 0.001), and biliary stenting (OR 2.49, 95% CI 1.53–4.04, *p* < 0.001) were associated with prolonged procedure duration. After multivariate analysis, these factors remained independently associated with prolonged procedure duration: late NKF (OR 11.49, 95% CI 5.84–22.62, *p <* 0.001), CBD sludge (OR 2.30, 95% CI 1.25–4.23, *p =* 0.008), PD cannulation (OR 4.46, 95% CI 2.32–8.56, *p <* 0.001), and biliary stenting (OR 1.95, 95% CI 1.03–3.67, *p =* 0.039) (Figure 2).

**TABLE 2:** Univariate and multivariate analysis of the possible risk factors for prolongation of overall duration of the procedure.

Variable	Univariate analysis		Multivariate analysis	
	Odds ratio (95% CIs)	*p*	Odds ratio (95% CIs)	*p*
Older age	1.00 (0.988–1.014)	0.837	—	—
Female sex	1.154 (0.745–1.787)	0.520	—	—
BMI	0.964 (0.917–1.013)	0.151	—	—
Late NKF (versus pNKF)	22.2 (12.079–40.800)	< 0.001	11.493 (5.839–22.620)	< 0.001
CBD diameter	0.959 (0.897–1.026)	0.228	—	—
Balloon dilation	0.870 (0.384–1.973)	0.740	—	—
CBD sludge	1.853 (1.174–2.925)	0.008	2.298 (1.248–4.232)	0.008
Stone number	1.062 (0.927–1.218)	0.382	—	—
Stone size	0.962 (0.903–1.024)	0.231	—	—
PD cannualtion	13.955 (8.027–24.259)	< 0.001	4.457 (2.320–8.562)	< 0.001
Successful stone extraction	0.918 (0.407–2.066)	0.836	—	—
Biliary stent	2.490 (1.533–4.044)	< 0.001	1.948 (1.033–3.673)	0.039

Abbreviations: BMI, body mass index; CBD, common bile duct; CI, confidence interval; NKF, needle‐knife fistulotomy; PD, pancreatic duct.

### 3.2 Cohort II (Patients Undergoing NKF With Complete Stone Extraction)

**FIGURE 3:**
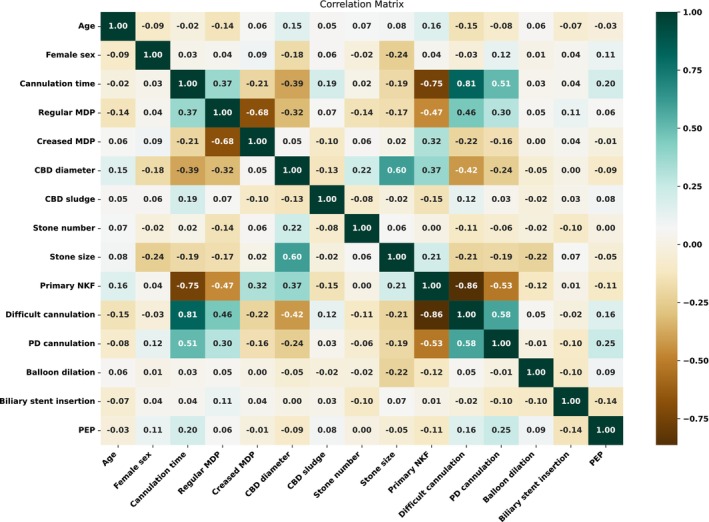
Correlation matrix of the variables in PS matched cohort with successful stone extraction. CBD, common bile duct; MDP, major duodenal papilla; NKF, needle‐knife fistulotomy; PD, pancreatic duct; PEP, post‐ERCP pancreatitis.

This cohort initially comprised 331 patients (50 with biliary stent, 281 without). Following 1:4 PSM, both groups consisted of 220 patients (44 with biliary stent, 176 without). Figure 3 shows the correlation matrix between variables in the matched cohort.

Table 3 shows the baseline characteristics, ERCP findings, and the adverse events in this cohort, before and after PSM Before PSM, the stented group was slightly older (59.7 years vs. 55.3 years; *p* = 0.094). After matching, this difference was further reduced (*p* = 0.334). Sex and BMI were similar between groups both before and after matching (*p* > 0.05). No significant differences were observed in papilla morphology between the groups before or after matching. However, the stented group had a higher rate of late NKF compared to the non‐stented group before matching (56.0% vs. 35.9%; *p* = 0.007); this difference was not observed after matching (*p* = 0.840). Cannulation time was significantly longer in the stented group before matching (252 s vs. 197 s; *p* < 0.001). After matching, this difference was no longer significant (*p* = 0.520). Similarly, the higher rate of difficult cannulations in the stented group before matching (64.0% vs. 40.2%; *p* = 0.002) was not observed after matching (*p* = 0.787). The CBD diameter was larger in the stented group before matching (13.5 mm vs. 11.3 mm; *p* < 0.001), but this difference was not significant after matching (*p* = 0.950). Balloon dilation was less frequently performed in the stented group before matching (88.0% vs. 95.4%; *p* = 0.039), with no significant difference remaining after matching (*p* = 0.162). The presence of CBD sludge and the number of stones were comparable between groups before and after matching (*p* > 0.05). The stented group had larger stones before matching (12.4 mm vs. 9.0 mm; *p* < 0.001). However, after matching, stone size differences were not significant (*p* = 0.426). The overall duration of ERCP was longer in the stented group before matching, with a higher proportion of procedures exceeding 30 min (46.0% vs. 23.5%; *p* = 0.003). After matching, the duration distribution was similar between groups (*p* = 0.523).

**TABLE 3:** Demographics, ERCP findings, interventions, and adverse events in patients with choledocholithiasis with successful stone extraction.

Variables	Before PS matching			After PS matching		
	With stent (*n* = 50)	Without stent (*n* = 281)	*p*	With stent (*n* = 44)	Without stent (*n* = 176)	*p*
Age (years)	59.7 ± 15.2	55.3 ± 17.1	0.094	53.5 ± 11.6	56.1 ± 16.7	0.334
Female sex	28 (56.0)	149 (53.0)	0.698	28 (63.6)	103 (58.5)	0.536
BMI (kg/m^2^)	25.5 ± 3.7	25.9 ± 4.8	0.593	26.7 ± 3.2	25.8 ± 4.9	0.287
Papilla morphology						
Regular	15 (30.0)	87 (31.0)	0.554	21 (47.7)	61 (34.7)	0.091
Small	0	3 (1.1)		0	3 (1.7)	
Protruding	7 (14.0)	63 (22.4)		2 (4.6)	37 (21.0)	
Creased	28 (56.0)	128 (45.5)		21 (47.7)	75 (42.6)	
NKF technique						
Primary	22 (44.0)	180 (64.1)	0.007	22 (50.0)	85 (48.3)	0.840
Late	28 (56.0)	101 (35.9)		22 (50.0)	91 (51.7)	
Cannulation time (seconds)	252 ± 112	197 ± 102	< 0.001	229 ± 122	217 ± 106	0.520
Difficult cannulation	32 (64.0)	113 (40.2)	0.002	22 (50.0)	92 (52.3)	0.787
CBD diameter (mm)	13.5 ± 3.9	11.3 ± 2.9	< 0.001	11.9 ± 2.54	11.9 ± 2.7	0.950
Balloon dilation	44 (88.0)	268 (95.4)	0.039	39 (88.6)	167 (94.9)	0.162
CBD sludge	16 (32.0)	85 (30.3)	0.804	15 (34.1)	54 (30.7)	0.663
CBD stone	57 (32.0)	214 (64.7)	< 0.001	56 (47.1)	65 (54.6)	0.243
Stone size (mm)	12.4 ± 5.2	9.0 ± 4.2	< 0.001	9.9 ± 3.7	9.2 ± 3.7	0.426
Stone number	2.4 ± 1.55	2.5 ± 2.11	0.795	2.0 ± 1.27	2.6 ± 2.2	0.227
PD cannulation	15 (30.0)	55 (19.6)	0.096	8 (18.2)	51 (29.0)	0.184
Duration of ERCP						
≤ 15 min	19 (38.0)	169 (60.1)	0.003	21 (47.7)	86 (48.8)	0.523
15–30 min	8 (16.0)	46 (16.4)		7 (15.9)	39 (22.2)	
> 30 min	23 (46.0)	66 (23.5)		16 (36.4)	51 (29.0)	
Adverse events						
PEP						
Overall	2 (4.0)	33 (11.7)	0.134	1 (2.3)	23 (13.1)	0.054
Mild	2 (4.0)	25 (8.9)		1 (2.3)	18 (10.2)	
Moderate	0	8 (2.8)		0	5 (2.9)	
Severe	0	0		0	0	
Bleeding	1 (2.0)	0	0.151	1 (2.3)	0	0.200
Cholangitis	2 (4)	2 (0.7)	0.110	2 (4.6)	2 (1.1)	0.179
Perforation	0	0	1.000	0	0	1.000
Stent migration	1 (2)	0	0.329	1 (2.3)	0	0.452

Abbreviations: BMI, body mass index; CBD, common bile duct; ERCP, endoscopic retrograde cholangiopancreatography; NKF, needle‐knife fistulotomy; PD, pancreatic duct; PEP, post‐ERCP pancreatitis; PS, propensity score.

Before matching, PEP occurred less frequently in the stented group (4.0% vs. 11.7%; *p* = 0.134), but after matching, this difference became borderline significant (2.3% vs. 13.1%; *p* = 0.054). Prophylactic biliary stenting reduced the incidence of PEP (RR = 0.17, 95% CI 0.02–1.25, *p* = 0.083). The number needed to treat was 9 (95% CI 6–25). There were no significant differences in bleeding and cholangitis rates between the groups, both before and after PSM (*p* > 0.05). One stent migration was observed in the stented group (2.0% before PSM, 2.3% after PSM). No perforations were observed in either group.

## 4 Discussion

This study assessed the hypothesis of whether biliary stenting after NKF could improve the safety of the ERCP and reduce its related adverse events. This hypothesis was evaluated in two stages: cohort I (which consisted of patients with choledochilithiasis regardless of stone extraction success) and cohort II (which was a subgroup of cohort I with successful complete stone extraction). The results revealed that after successful extraction of CBD stones using NKF techniques, insertion of a biliary stent could improve the safety of the procedure by reducing PEP rates.

NKF is an advanced cannulation technique usually used after failure of the conventional wire‐guided technique. Recent studies also suggest using this technique as the initial cannulation approach in certain papilla morphologies [17, 18]. This technique is gaining popularity as many endoscopists believe it is associated with a lower rate of PEP, owing to its ability to reduce thermal injury to the pancreatic orifice and also a lower rate of inadvertent PD cannulation [19]. Moreover, compared to other rescue cannulation techniques, including needle knife papillotomy, it is associated with a lower rate of adverse events [20]. Nonetheless, the increasing adoption of this technique is not solely attributed to its lower PEP rate; it is also associated with a high rate of successful biliary cannulation. Meta‐analyses have estimated a success rate of biliary cannulation of 95.7% for primary NKF and 91.7% for late NKF [21]. Due to this high rate of successful cannulation and low rate of adverse events, NKF is evolving into a widely utilized technique that every endoscopist should master.

The most concerning issue following NKF is its risk of perforation. A careless incision in NKF may lead to retroperitoneal perforation, a catastrophic adverse event that is associated with a high mortality rate and may require emergency surgery [22]. The rate of perforation following NKF has been reported to be up to 1.5% [23], which is significantly higher than that of conventional cannulation techniques, with an approximate rate of 0.4% [24]. The impact of an endoscopist's level of experience on NKF outcomes is a topic of debate. A retrospective study by Han et al. [25] investigated the impact of endoscopist experience on the outcomes of NKF and reported that NKF is an effective and safe technique even when it is performed by less experienced endoscopists. In the present study, we were unable to investigate this, as only three experienced endoscopists performed the procedures. However, regarding perforation, a more critical point in this study is that due to the rare occurrence of perforation, we were unable to investigate the impact of biliary stents on preventing periampullary and biliopancreatic perforations. In this study, three patients who experienced periampullary perforation during cannulation prior to stone extraction were excluded from the study, and delayed perforation was not observed in any of the patients. Theoretically, biliary stents cover the CBD surface and may prevent delayed perforations. However, examining the role of biliary stents in preventing delayed perforations was not feasible in this study due to the rarity of perforation occurrence; addressing such a question would require a significantly larger sample size.

This study revealed that prophylactic biliary stenting after NKF may improve the safety of the procedure after stone clearance of the CBD. To the best of our knowledge, there is no study focusing on biliary stenting after the NKF. Nonetheless, two recent studies investigated the effect of biliary stenting on the adverse events and recurrence of CBD stone after stone clearance of the CBD. A retrospective large‐scale study was performed by Chandan et al. [26] using the United States Readmission Database. They reported that prophylactic biliary stenting after stone clearance resulted in a higher rate of PEP (0.05% vs. 0.03%), longer length of hospitalization, and total hospitalization charge. A randomized clinical trial by Sasani et al. [27] reported that prophylactic biliary stenting after stone clearance was associated with a higher rate of adverse events (14.7% vs. 0%), while there was no significant difference in stone recurrence within 3 months (20.6% vs. 9.4%). However, it should be noted that the cannulation technique was the focus of none of these studies; both studies included a variety of cannulation techniques including conventional transpapillary, fistulotomy, and papillotomy techniques. Moreover, contrary to Sasani et al. there are reports of a decrease in the recurrence rate of CBD stones after prophylactic biliary stenting following stone clearance [28, 29].

Despite the advances in preventive interventions for PEP, such as PD stenting, this adverse event continues to be the most frequent adverse event associated with ERCP [30]. Therefore, preventive approaches must be optimized based on the target population. The findings of this study suggest that prophylactic biliary stenting after stone clearance may reduce the rate of PEP. After stone removal, spasm or edema of the sphincter of Oddi can result in papillary obstruction, increasing PD pressure and promoting enzyme activation. A biliary stent creates a scaffold that maintains patency at the papilla and ensures continued bile and PD drainage even without stenting of the PD. This is particularly relevant because bile and PDs share a common anatomical path at the papilla; stent placement into the bile duct maintains indirect patency with the common channel, allowing for pancreatic outflow. Additionally, by decompressing the biliary tree, the stent reduces biliary pressure, with a possible relieving effect also on pressure within the adjacent PD, especially where ducts are anatomically close or share a common channel. This two‐fold mechanism minimizes ductal hypertension, an important factor for PEP pathogenesis. The stent, secondly, prevents reflux of bile back into the PD, a phenomenon that can lead to pancreatitis by activation of pancreatic enzymes in the ductal network. By ensuring unimpeded bile flow and decreasing retrograde pressure gradients, the stent decreases the likelihood of bile‐pancreatic reflux. These mechanisms combined—maintained papillary drainage, preservation of the common channel, biliary decompression, and prevention of reflux—address the mechanical and hydrodynamic disruptions that cause PEP. While PD stenting remains the gold standard for PEP prevention in high‐risk cases, biliary stenting offers a useful option in some circumstances, such as difficult biliary cannulation or significant papillary trauma during NKF.

In summary, it appears that for patients with CBD stones, the combination of NKF and biliary stenting can be beneficial, regardless of whether stone extraction is successful. For patients with unsuccessful stone extraction, as recommended by ESGE and American Society for Gastrointestinal Endoscopy (ASGE) guidelines, biliary stenting facilitates biliary drainage and aids subsequent stone removal [3, 4, 31]. In patients with successful stone extraction, as demonstrated by the results of this study, biliary stenting may enhance procedural safety, particularly by reducing the rate of PEP. However, the results of the study must be considered in light of several limitations. The most significant limitation was the small number of participants, especially in the subgroup with successful stone extraction and biliary stenting; although PSM was performed to make the groups more comparable. Second, the low incidence of adverse events, such as PEP, made it impossible to use more sophisticated statistical analyses, allowing for more precise group comparisons and successful control of confounding factors. This low incidence of adverse events, combined with small sample size, may have limited the statistical power to detect significant differences. While reduction in PEP with stenting did not reach statistical significance, the observed effect sizes may still be clinically meaningful. Third, due to the rare occurrence of perforation, the study was unable to assess the impact of biliary stenting on the prevention of delayed perforation, arguably the most important outcome, which, given the rarity of this event, would require a sample size exceeding 20 000 patients, making it currently impractical. Fourth, the exclusion of patients requiring prophylactic PD stenting leaves unanswered whether simultaneous biliary and pancreatic stenting might further reduce the rate of PEP in high‐risk patients. Additionally, since all procedures and NKFs were performed by experienced endoscopists, the generalizability of these findings to less experienced endoscopists may be limited. Lastly, as with any retrospective study, there is a risk of confounding variables and selection bias impacting the results. Large‐scale randomized clinical trials could provide further insight into this topic.

## 5 Conclusions

To the best of our knowledge, this is the first study to investigate the impact of prophylactic biliary stenting after NKF. The results suggest that prophylactic biliary stenting after NKF may improve the safety of the procedure after stone clearance of the CBD, particularly by reducing the incidence of PEP. Further large‐scale randomized trials are needed to confirm these findings.

## Conflicts of Interest

The authors declare no conflicts of interest.

## Data Availability

The data that support the findings of this study are available on request from the corresponding author. The data are not publicly available due to privacy or ethical restrictions.
